# Physicochemical and Biomechanical Stimuli in Cell-Based Articular Cartilage Repair

**DOI:** 10.1007/s11926-014-0493-9

**Published:** 2015-04-02

**Authors:** Holger Jahr, Csaba Matta, Ali Mobasheri

**Affiliations:** 1 Department of Orthopaedic Surgery, University Hospital RWTH Aachen University, Pauwelsstraße 30, 52074 Aachen, Germany; 2 The D-BOARD European Consortium for Biomarker Discovery, Surrey, UK; 3 Department of Veterinary Preclinical Sciences, School of Veterinary Medicine, Faculty of Health and Medical Sciences, University of Surrey, Duke of Kent Building, Guildford, Surrey GU2 7XH UK; 4 Department of Anatomy, Histology and Embryology, Faculty of Medicine, University of Debrecen, Nagyerdei krt. 98, Debrecen, 4032 Hungary; 5 Arthritis Research UK Centre for Sport, Exercise and Osteoarthritis, Arthritis Research UK Pain Centre, Medical Research Council and Arthritis Research UK Centre for Musculoskeletal Ageing Research, University of Nottingham, Queen’s Medical Centre, Nottingham, NG7 2UH UK; 6 Center of Excellence in Genomic Medicine Research (CEGMR), King Fahd Medical Research Center (KFMRC), King AbdulAziz University, Jeddah, 21589 Kingdom of Saudi Arabia

**Keywords:** Articular cartilage, Cartilage repair, Regenerative medicine, Autologous chondrocyte implantation, Chondrocyte metabolism, Functional tissue engineering, Cartilage bioengineering, Mesenchymal stem cell, Intracellular signaling pathways

## Abstract

Articular cartilage is a unique load-bearing connective tissue with a low intrinsic capacity for repair and regeneration. Its avascularity makes it relatively hypoxic and its unique extracellular matrix is enriched with cations, which increases the interstitial fluid osmolarity. Several physicochemical and biomechanical stimuli are reported to influence chondrocyte metabolism and may be utilized for regenerative medical approaches. In this review article, we summarize the most relevant stimuli and describe how ion channels may contribute to cartilage homeostasis, with special emphasis on intracellular signaling pathways. We specifically focus on the role of calcium signaling as an essential mechanotransduction component and highlight the role of phosphatase signaling in this context.

## Introduction

The unique biomechanical properties of articular cartilage are attributed to the structure, composition, and organization of its extracellular matrix (ECM) macromolecules. The cartilage ECM is mainly composed of a collagen fiber network (type II collagen with type IX and XI) and large aggregating proteoglycans (PGs) entrapped within. The fixed negative charges on the glycosaminoglycan (GAG) side chains of PGs attract counteracting ions, which drive the movement of large amounts of osmotically obliged water into the matrix [1]. The high osmotic milieu and the tissue swelling maintain the hydrostatic pressure and viscoelastic properties of cartilage. The highly sulfated GAG side chains of PGs, through attracting mobile cations, are responsible for the characteristically high negative fixed-charge density (FCD) [2]. Intertwined collagens and PGs combine to create the tissue rigidity by entrapping solutes and water, giving cartilage its unique biomechanical properties, to withstand large compressive and shear forces without failing [3]. Articular cartilage absorbs stresses generated during joint loading and contributes to joint lubrication [1, 4]. An intact collagen network restricts swelling and, in combination with sulfated GAGs, determines the osmotic pressure (OP) of the extracellular fluid around chondrocytes, which ranges from 350 to 480 mOsm in healthy cartilage [5].

The electrochemical properties of articular cartilage arise from the flow of “free” electrolytes (e.g., Na^+^, K^+^, Ca^2+^) passing the relatively “fixed” FCD (e.g., SO_4_^2−^) distributed along the PGs [6] resulting in electrokinetic phenomena and charge-dependent osmotic swelling pressures (i.e., Donnan osmotic pressure) [7–9]. The FCD permits tissue hydration, charged species transport, and other electrochemical responses [10]. Mow et al. postulated that the ECM is a mechanical signal transducer, receiving loading as input to generate an output of multiple biophysical signals [11].

Interestingly, reports on physiologically relevant values of tensile or shear forces in natural cartilage or in tissue-engineered constructs are sparse [12], as is the knowledge about the molecular identity of the sensory components and signaling apparatus that convert various environmental forces (e.g., deformation, shear stress and fluid flow, hydrostatic pressure (HP), and extracellular ionic milieu (i.e., OP) as well as magnetic and electric forces), into cellular responses. We provide a brief overview of how these forces might be exploited to facilitate cartilage regeneration, with special emphasis on intracellular signaling, which is often understudied in the context of cartilage bioengineering.

## Cartilage Pathologies

Traumatic local damage [13–15], usually in younger patients, and whole joint erosion, as in osteoarthritis (OA) [16] in the elderly, are challenging areas of regenerative orthopedics [17]. To date, there is no successful targeted therapy that would halt or even reverse OA progression; current management including inflammatory medications, total joint replacements, or analgesics only allow palliative treatment [18•]. There is a pressing need for targeted treatment options, ideally at the early, asymptomatic stages of the disease. The earliest signs of articular cartilage degeneration during OA are net depletion of PGs [10, 19–21], subsequent loss of the collagen network [22], and diminished intrinsic compressive stiffness, affecting chondrocyte deformation, metabolic activities, and electromechanical events within cartilage under body load [10, 23]. Severity-dependent catabolic events during the course of OA reduce extracellular osmolarity, resulting in reduced viscoelastic tissue properties, corresponding biomechanical inferiority [5, 24], and eventually increased deformation of cartilage under mechanical load. Elevated levels of inflammatory mediators that promote matrix degradation may also accompany these changes.

## Regenerative Approaches

Autologous chondrocyte implantation (ACI) [25] surpasses existing procedures for treating focal defects, but is unable to fully restore functional hyaline cartilage *ad integrum*. A potentially better procedure for structurally repairing symptomatic cartilage defects in the knee is characterized chondrocyte implantation (CCI) which has a more favorable outcome compared to microfracturing [26].

A major challenge is the complex zonal structure of cartilage tissue, which is important for its load-bearing properties [27–29]. The sparse available data indicate that mechanical properties significantly vary between articular cartilage zones [30–32]. The lack of mechanical homogeneity may be important for mechanosensation, signal transduction, and chondrocyte phenotypic stability.

Another major challenge of present tissue engineering strategies for cartilage repair is the limited integration of the constructs into the surrounding host tissue [33], often resulting in local cell death at the defect margins [34]. The goal, therefore, is to create tissue functionality prior to implantation by enhancing the rate and quality of tissue growth through creating in vivo-like conditions in vitro. Various environmental stimuli for promoting cartilage regeneration are discussed in the following sections.

### Stimulating Functional Cartilage Tissue Engineering

#### Electrical Stimulation

Mechanoelectrical transduction phenomena occurring naturally within the cartilage due to the FCD have prompted the development of experimental electrical stimulation protocols for therapeutic cartilage repair [35, 36]. Therapeutic devices involving electrical stimulation are increasingly entering the clinical market [37], despite rather discouraging early results [38, 39]. More recently, Brighton et al. observed anabolic effects [40] that may hold potential to treat osteoarthritic lesions [18•]. Our current appreciation of underlying molecular mechanisms, however, is rudimentary.

#### Magnetic Stimulation

Since its FDA approval in 1979, pulsed electromagnetic field (PEMF) therapy has been widely used in orthopedics to treat poorly healing fractures [41, 42•]. Although the biology of how PEMF stimulates bone formation is only partially understood [43] and may originate from stimulating progenitor cell differentiation [44], its clinical use has provided a rationale for applying (P)EMF in musculoskeletal tissue engineering [45, 46].

Data from randomized controlled trials now suggest that PEMF improves clinical scores and function even in patients with knee OA [47]. While (low-frequency) PEMF therapy barely influences the biosynthetic activity of human OA chondrocytes in vitro [48], it increases PG release in alginate culture [49]. PEMF increases anti-inflammatory effects in the human costal chondrocyte cell line T/C-28a2 [50], and, like IGF-1, it augments chondroprotective anabolic activities such as PG synthesis in human OA cartilage explants, possibly by counteracting the effects of IL-1β in early stages of OA [51]. A study in ovariectomized rats, aiming at simulating postmenopausal osteoarthritis, reported an interesting systemic effect of PEMF therapy on estrogen metabolism that reduced apoptosis and matrix metalloproteinase (MMP)13 expression in knee joint cartilage [52]. However, since current evidence for PEMF as a disease-modifying OA therapy is still weak, further studies are needed to elucidate its molecular basis.

#### Mechanical Stimulation

In comparison to the rather limited evidence for effects of magnetic and electrical stimulation, a vast body of studies have described the consequences of mechanical stimulation on articular cartilage or chondrocytes in tissue engineering strategies [18•]. We review mainly cellular responses of chondrocytes and aspects closely related to osmotic stress, such as compression-induced changes in HP and OP, as both are essential for stimulating chondrocyte physiology and useful for manipulating chondrocyte metabolism and phenotype [53].

Mechanical stimulation is an important regulator of chondrocyte metabolism that is required for maintaining normal cartilage matrix properties [54, 55] and a well-established cue for improving mechanical properties of tissue-engineered cartilage [27], as reviewed by Vunjak-Novakovic et al. [56] and Lee et al. [57]. A plethora of bioreactors have been developed in which mechanical forces are applied via compression, HP, shear, multimodal compression and shear, vibration, bi-axial tension, and friction [18•]. While static loading of tissue-engineered cartilage constructs, in general, results in suppression of ECM biosynthesis, intermittent dynamic loading is usually beneficial and increases the biosynthetic activity of chondrocytes. The cellular response to mechanical stimuli always depends on magnitude, frequency, and duration of the stimulus, as well the relative timing of the loading, the culture period, and the subpopulation of chondrocytes. Importantly, the balanced activities of catabolic and anabolic factors may be needed to stimulate native-like ECM synthesis [58, 59].

##### Intrinsic Mechanical Stimulation and Substrate Properties

Cyclic compression is required for chondrogenesis [60], while its impact on the intrinsic material properties of cartilage is an underappreciated aspect in tissue engineering. Using surface topography, stiffness, or patterns to induce mesenchymal stem cell (MSC) proliferation or differentiation [61] holds a lot of potential for enhancing musculoskeletal regeneration [62•].

Our understanding of how cells sense the stiffness of ECM or biomimetic substrates is rudimentary. Emerging mechanisms of biophysically induced signals include focal adhesions and cytoskeletal or Rho GTPase functions [63, 64]. Local matrix stiffness can determine cell development, differentiation, and regeneration through adhesion complexes [64] with the actin–myosin cytoskeleton generating intrinsic contractile forces by “sensing” substrate properties via pre-stretching through actin stress fibers; linking integrin transmembrane receptors to ECM in mechanosensation enables primary cells to alter their function in response to exogenous forces [65] or oxygen tension and local cell density [66]. Initial attempts suggest the feasibility of creating 3D stiffness gradients in hydrogels [67] to re-differentiate chondrocytes. By manipulating substrate elasticity and adhesion density [68], stiffness may affect proliferation and RGD adhesion site density during cellular differentiation. While the banding periodicity of collagen fibers in the ECM is 67 nm [69] and the RGD epitopes of fibronectin fibers are ≥73 nm [70, 71], cells are clearly sensitive to changes in interparticle spacing of about 1 nm over a cell length [63],

That MSC fate can be re-directed, even after weeks, by switching the biophysical microenvironment [72••] holds promises for several cartilage-related tissue engineering applications. In contrast to generally unfavorable static compression, static pre-stretching of biomaterials may beneficially alter (stem) cell behavior [73] through regulation of epigenetic events [74]. A meshwork of intermediate filaments and lamins physically links chromatin to the cytoskeleton-mediated extracellular signal reception [75, 76]. Mechanical forces arising from matrix rigidity and nanotopography can physically affect the structural organization of the nucleus [77], possibly directly altering gene expression and mechanical properties [63].

In combination with mechanical stimulation, incorporation of chemical groups such as sulfates may improve chondrocyte proliferation while inhibiting hypertrophic differentiation [78, 79]. By using intrinsic biomaterial cues to stimulate migration, cell-seeded scaffolds appear promising for cartilage repair. Similar to MSCs, chondrocytes respond to HP, fluid flow (FF) and the accompanying shear stress, substrate strain and stiffness or topography, and electromagnetic fields [80]. Fully synthetic hydrogels can provide independent control over physical and adhesive properties [81] for use in cartilage regenerative medicine [82].

#### Osmotic Stimulation

Chondrocytes in cartilage represent cells under pressures of different natures, like deformation, hydrostatic pressure, extracellular ion composition (i.e., OP), and streaming potential (i.e., FF) [54]. Further, the concept is generally accepted that matrix turnover by chondrocytes is influenced by changes to the intracellular composition (e.g., cell volume, pH, and ionic content). The pericellular microenvironment functions in situ to mediate the chondrocyte (or chondron) responses to physicochemical changes associated with joint loading [83]. During compression-induced changes in OP, the pericellular matrix exerts important functions through amplifying cell volume changes [84]; such findings argue in favor of using chondrons, rather than isolated chondrocytes, for osmo-induced cartilage tissue engineering.

About 15 years ago [11], Mow et al. described the sometimes counter-intuitive effects of flow-induced compression of the ECM and hypothesized that this friction-drag effect is likely of major importance for fluid flow through the ECM. Changes in HP and OP are essential for chondrocyte physiology and useful for manipulating their metabolic function and phenotype [53]. Applying controlled HP to cartilage or chondrocytes can be technically challenging [53], while OP is robustly defined as chemical. Unlike OP, tonicity is influenced only by solutes that cannot cross the cell membrane. Although chemical loading (i.e., OP) and mechanical loading (i.e., HP) may not be exactly equivalent [85], the combination of HP and OP produce gene expression profiles different from those with OP alone, each stimulus by itself often results in similar effects such as the stimulation of sulfated GAG synthesis [53].

As OP is a state quantity, it changes during compressive joint loading and off-loading; with zone-dependent concentrations of sulfated GAGs causing OP gradients in articular cartilage [86]. In each zone, chondrocytes are subject to different HPs and OPs due to weight bearing and joint loading [53, 32]. Applying HPs from 0 to 0.5 MPa at 0.5 Hz and OPs from 300 to 450 mOsm can upregulate anabolic and catabolic molecules in all three major zones in a descending order of magnitude from the surface to the deep zone. Interestingly, HP off-loading maintains anabolic messenger RNAs (mRNAs) and reduces catabolic mRNAs, while high OP retains mainly catabolic mRNAs [53]. Superficial zone-derived cells are most sensitive to changes in HP or OP [53], which may explain discrepancies between chondrocytes isolated from “normal” and OA cartilage. The effects of OP on viscoelastic and physical properties of chondrocytes are well described [87]. Tonicity enhancer binding protein (TonEBP, also known as nuclear factor of activated T cells (NFAT)5) stimulates multiple cellular pathways for adaptation to osmotic stress [88, 89] and organic osmolyte-dependent and independent pathways [90]. Physiological and pathophysiological stimuli such as cytokines, growth factors, receptor and integrin activation, contractile agonists, ions, and reactive oxygen species have been implicated in the positive regulation of TonEBP expression and activity in diverse cell types [91].

Under standard FCS-containing expansion culture conditions, proliferation of human chondrocytes seems to be unhampered up to physiological osmolarity levels (i.e., ∼350–400 mOsm) [92, 93]. Proteomic analysis of serum-free expanded chondrocytes has confirmed a cutoff threshold of about 350 mOsm, above which cell cycle progression and proliferation appears compromised [94, 95].

##### Molecular Aspects

The response to osmotic loading seems to depend on the nature of the osmotic stimulation and the chondrocyte phenotype, which is related to passage number and pathological state [96]. Osmotic loading differentially regulates SOX9 and COL2A1 mRNA stability posttranscriptionally [97]. In nucleus pulposus cells, NFAT5 [98], together with intracellular Ca^2+^ [99] and MEK/extracellular signal-regulated kinase (ERK) signaling [100], control cell function, survival, and sulfated GAG synthesis [101, 102]. Hyperosmotic stress induces volume changes and Ca^2+^ transients in chondrocytes by transmembrane ion channels, phospholipids, and G-protein coupled pathways [103].

Pritchard et al. [104] found that IL-1α alters the normal volumetric and Ca^2+^ signaling response of porcine chondrocytes to OP through mechanisms involving F-actin remodeling and Rho GTPases. Human OA chondrocytes have a more positive membrane potential (i.e., −26 ± 4 mV) than healthy cells and show reduced [Ca^2+^]_o_ independent protein kinase C (PKC)α-mediated hyperpolarization upon hyperosmotic stimulation [105].

Osmotic loading is known to modulate chondrocyte height, width, and volume in situ, and OP may modulate cell shape in accordance with the primary collagen fibril direction [106], as well as altering nuclear size and shape [107]. Interestingly, osmotic sensitivity of nuclear shape and volume appeared to be independent of the actin cytoskeleton. While compression (and thus increased OP) reduces the ECM, cellular, nuclear, rER, and mitochondrial volumes, the Golgi apparatus seems relatively resistant to intraorganelle water loss [108]. This may, at least partially, explain some of the observed posttranscriptional effects of OP [97].

##### Clinical Relevance

Chondrocyte shrinkage by raised hyperosmotic pressure (≥480 mOsm) may protect cells. While most cell-based chondral repair strategies aim at re-differentiation of routinely expanded, dedifferentiated chondrocytes, van der Windt et al. showed that dedifferentiation can be delayed by harvesting and expanding cells under elevated (i.e., physiological, 380 mOsm) osmolarity [92]. Interestingly, combining physiological osmolarity with inhibition of calcineurin activity can increase the expression of anabolic genes and suppress catabolic genes, as well as hypertrophic markers, in human OA and “normal” chondrocytes [93] and may be a promising strategy for improving cell-based chondral defect repair. The clinical potential of applying osmolarity to improve the chondrocyte phenotype is hard to predict from present in vitro data, given the depth zone dependence of osmotic responses [109] and the current clinical practice of harvesting chondrocytes irrespective of their original zonal location.

The effects of OP, to a certain extent, also depend on the culture model: in alginate, higher proliferation rates, with diminished sulfated GAG production, were found at 280 mOsm [110]. Of note, the pH_i_ is also osmolarity-dependent and its contribution to sulfated GAG production remains speculative.

Finite element modeling showed that charged tissues (or synthetic matrices) always support larger loads than uncharged tissues. This load support derives from three sources: intrinsic matrix stiffness, HP, and OP [111].

### Regulation of Phosphatases by Chemo- and Biomechanics

#### Calcineurin as a Potential Target Molecule

A precisely set balance between the activities of protein kinases and phosphoprotein phosphatases is crucial to regulating chondrogenesis and maintaining the chondrocyte phenotype. All of the major protein kinase families, including protein kinase A (PKA), PKC, mitogen-activated protein kinase (MAPK), and CaMK, as well as all major protein phosphatases (PP1, PP2A, and PP2B) play fundamental roles in molecular regulation in chondrocytes [112]. These signaling pathways eventually converge on targets that are involved in defining the chondrocyte phenotype, and they regulate cell shape, proliferation, differentiation, and gene expression (via transcriptional regulators such as Sox9, cAMP response element binding protein (CREB), and NFAT; see details below).

The Ca^2+^-dependent serine/threonine phosphoprotein phosphatase calcineurin (Cn; also known as PP2B) has been identified as a potential target to improve the chondrocyte phenotype. The Cn inhibitor FK506 (also known as Tacrolimus) increases the expression of chondrogenic markers during in vitro expansion in hypoosmotic culture medium [113]. Isolation and expansion of adult human articular chondrocytes in culture medium of physiologic osmolarity (i.e., 380 mOsm) improves chondrogenic marker gene expression and ECM production through NFAT5 [92]. Interestingly, FK506 within the range of 0.1 and 1000 ng/mL increased not only COL2A1 but also COL10A1 expression, while in human OA cells FK506 suppressed the osmolarity-induced COL10A1 expression [93]. Generally, similar anabolic and anti-hypertrophic effects were observed in ex vivo cartilage explant cultures and non-OA chondrocytes. Similar data were reported with alternative Cn inhibitors (i.e., cyclosporine A, CsA) in human cells [114] and in the murine AT805-derived chondrogenic ATDC5 cell line [115], where FK506 increased PG content in a dose-dependent manner without elevating alkaline phosphatase (ALP) activity.

The exact mechanism underlying the effects of Cn inhibition under different osmolarities is not yet understood, but Cn is known to induce FGF18, which can suppress noggin and facilitate BMP-related chondrogenesis-like effects [116]. This pathway may involve, among others, NFAT4-mediated induction of BMP2 [117]. FK506, but not CsA, induces ATDC5 differentiation [118], suggesting that FK506 promotes chondrogenic differentiation, at least partly, by Cn-independent signaling routes. Since FK506 has been proven effective and safe as an anti-rheumatoid arthritis drug [119, 120], this approach may improve cell-based chondral repair strategies by interfering with adverse inflammatory or immune cell-mediated effects.

#### Cn–NFAT Signaling in Cartilage Pathologies

Cn regulates the activity of NFAT family members in a specific and Ca^2+^/calmodulin-dependent manner [121]. For a detailed overview of this vertebrate-specific phosphatase in chondrocyte physiology, the reader is referred elsewhere [112]. NFATs have arisen from an ancient precursor with a Rel domain, and Cn–NFAT signaling may be an essential process during vertebrate development [122].

After the original study by Glimcher’s group had shown that all four NFATc1–4 proteins are expressed in the cartilage [123], Greenblatt and colleagues recently expanded the earlier studies of Ranger et al. and Wang et al. [123, 124] by demonstrating essential functions of NFATs (i.e., NFATc1 and NFATc2) in articular cartilage homeostasis [125]. NFATs have the potential to link many extracellular signals to the nuclear transcriptional machinery [126].

Greenblatt’s cartilage-specific NFATc1 and NFATc2 double mutant mice showed accelerated cartilage degeneration and expression of OA markers, such as increased expression of genes encoding proteases involved in ECM degradation such as MMP13, ADAMTS-5, and hypertrophic chondrocyte markers, including COL10A1, and reduced expression of Sox9 and PRG4, encoding lubricin. Intriguingly, NFATc1 protein expression is restricted to the superficial zone of articular cartilage, and its mRNA expression is reduced around cartilage lesions in human osteoarthritic patients [125]. A number of earlier in vitro studies suggest that NFAT signaling may also induce catabolic genes such as ADAMTS4 and 9 in chondrogenic cells [127, 128], which are findings contradictory to the protective roles observed in vivo. While NFATc3 seems less important for cartilage homeostasis, it may still be relevant in chondrogenesis [117]. Most notably, multiple pathways co-regulate the subcellular localization of the four Ca^2+^-dependent NFAT proteins (NFATc1–4). In contrast, osmotic stress, rather than Ca^2+^ signaling primarily regulates the more distantly related fifth family member NFAT5, as discussed above.

A recent study showed that lentiviral shRNA-mediated Nfatc2 knockdown in articular chondrocytes in vitro largely matches the in vivo phenotype and also upregulates pro-inflammatory cytokines [129]. In tracheal cartilage, Ca_V_3.2 T-type Ca^2+^ channels may be involved in Cn–NFAT-dependent modulation of Sox9 expression [130••]. A previous study using other Cn inhibitors such as CsA had already suggested the participation of Ca^2+^ channels [131].

Pharmacological inhibition of Cn by FK506 promotes chondrogenic marker expression in dedifferentiated human adult chondrocytes, probably through upregulation of TGFβ1 [113]. NFAT activity seems tightly regulated by upstream signaling pathways: both activators (Cn) and inhibitors (e.g., GSK-3) can link a large number of mechanical and biochemical stimuli to this protein family, but few extracellular regulators of NFAT activity in chondrocytes have been identified to date. Not surprisingly, pharmacological inhibition of GSK-3β signaling increases cartilage degeneration in rats [132], while FK506 in the same species protects the collagenous ECM of articular cartilage against osteoarthritic wear-and-tear erosion [133]. At present, it is not clear whether these effects are due to altered NFAT activity or other pathways affected by the inhibitors of Cn (CsA or FK506) or GSK (GIN). Earlier studies suggest a link between osmolarity-induced signaling pathways such as MAPK or Ca^2+^ signaling and Cn–NFAT signaling, which may be integrated through NFAT5. NFAT activity and expression in chondrocytes in vitro seems also to be dependent on both Notch and Wnt5a signaling, at least in growth plate chondrocytes [134, 135], and its relevance for articular chondrocytes, especially in vivo, remains to be shown.

Overall, in vivo and in vitro data from mice and humans strongly suggest a dynamic control of NFATc2 expression in articular cartilage and a crucial role of NFAT family members in cartilage homeostasis and joint health. NFATs might be involved in distinguishing articular from growth plate chondrocytes, the origins of which are still not understood completely [136]. Novel small molecular compounds with higher specificities may make NFATs potential therapeutic targets for cartilage regenerative medicine and anti-osteoarthritic treatment regimes.

#### The Role of PKA and PP2A in Mechanical Stimulation and the Chondrocyte Phenotype

A main function of articular cartilage is to absorb shock during joint movements. Chondrocytes are sensitive to mechanical load, one of the most physiological stimuli that trigger the activation of key signaling molecules. Although appropriate mechanical stimuli are essential for limb development [137], differentiation of MSCs [138], and cartilage regeneration during OA [139], mechanotransduction pathways in differentiating or mature chondrocytes are still incompletely understood [140•].

Although mechanosensitive ion channels, primary cilia, and the actin cytoskeleton have all been implicated as mechanosensors in chondrocytes, downstream pathways are even less well characterized. Of the major signaling pathways, integrins and focal adhesion kinases (FAKs), the ERK, and the PI 3-kinase/Akt pathways have been reported [141]. It is of note that activation of the cAMP–PKA–CREB axis following mechanical stimuli has been documented in different models [142]. PP2A also plays a regulatory role in p38 MAPK activation during cyclic strain [143]. Oscillating mechanical load promotes chondrogenesis and stimulates cartilage ECM production in chicken limb bud-derived micromass cultures, and the observed effects can be attributed to the activation of PKA/CREB–Sox9 signaling and concurrent inhibition of the PP2A pathway [144]. Here, we propose that increased PKA activity results in enhanced Sox9 and CREB phosphorylation and nuclear translocation; these in turn facilitate chondrogenic differentiation and ECM matrix production. Given that PP2A is a negative regulator of chondrogenesis and balances the effects of PKA by dephosphorylating many common targets, its reduced activity further enhances the chondrogenesis-promoting effects of mechanical stimulation in this model. Interestingly, previous data also indicated a direct interplay between PKA and PP2A during chondrogenesis [145] and strongly support the important role of reversible protein phosphorylation in establishing and maintaining the chondrocyte phenotype.

Serially passaged articular chondrocytes, deprived of their ECM, rapidly lose their characteristic phenotype. Signaling events that control the re-differentiation of dedifferentiated chondrocytes have only partially been analyzed. Chondrocyte re-differentiation in micromass cultures may be mediated by PKC-dependent ERK1/2 regulation, whereas chondrocyte dedifferentiation is under a separate control by PKCα and ERK1/2 [146]. In a different study, p38 MAPK along with PKCα activity was reported to be essential for chondrocyte re-differentiation [147]. Since cyclic hydrostatic pressure upregulates cartilage-specific gene expression during re-differentiation of dedifferentiated bovine articular chondrocytes [148], one can speculate that mechanical load-induced activation of protein kinases and/or phosphatases may be responsible, at least partially, for these effects.

### Calcium Signaling Is an Essential Component in Mechanotransduction Pathways in Differentiating and Mature Chondrocytes

Intracellular Ca^2+^ signaling and changes in cytosolic Ca^2+^ concentration are closely related to cell proliferation and differentiation in chondroprogenitor cells, and Ca^2+^ release from intracellular stores and influx through plasma membrane ion channels are key factors controlling chondrogenesis [149]. Various chondrocyte plasma membrane ion channels appear to be regulated by mechanical stimuli, such as the big conductance Ca^2+^-activated K^+^ channel (BK-like channel) [150] or the transient receptor potential vanilloid 4 (TRPV4) cation channel [151••]. Mechanical load-induced Ca^2+^ influx and subsequent alterations in Ca^2+^ signaling have been documented in chondrocytes upon both compressive loading and HP [152]. Furthermore, cyclic compression is known to modulate cartilage matrix synthesis and catabolism through an autocrine/paracrine purinergic pathway; compression-induced ATP release evokes Ca^2+^ transients via activation of P2X and P2Y receptors that cause a combination of extracellular Ca^2+^ influx and intracellular Ca^2+^ release in agarose-embedded chondrocytes [101]. How exactly Ca^2+^ signaling is coupled to mechanosensation in chondrocytes remains an open question.

A promising candidate for a mechanosensory organelle on chondrocytes is the primary (non-motile) cilium, first identified on articular chondrocytes almost 40 years ago [153]. Tissue compression during joint loading can lead to deformation of the cilium, which in turn may trigger signaling involved in mechanotransduction pathways. Indeed, various extracellular matrix receptors including integrins, as well as osmo- and mechanosensitive ion channels including TRPV4, are known to be present on its surface [140•]. In particular, the primary cilium is necessary for compression-induced ATP release and Ca^2+^ signaling via P2X and P2Y purinergic receptors, inducing aggrecan mRNA expression and sulfated GAG secretion in a 3D chondrocyte culture system [154]. These findings suggest that the primary cilium does not act as the initial mechanosensor in that model, leaving several open questions regarding its specific role in chondrocyte mechanosensation.

## Conclusions

**Fig. 1:**
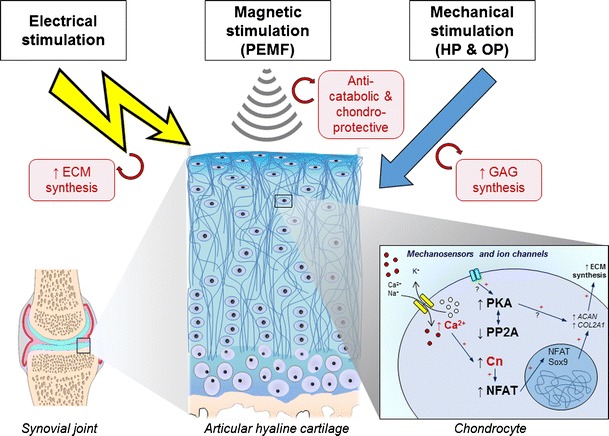
The effects of electrical, magnetic, and mechanical stimulation on articular cartilage

The effects of electrical, magnetic, and mechanical stimulation on articular cartilage are summarized in Fig. 1. Data are accumulating regarding the molecular identity of the sensors and the mechanotransduction signaling apparatus in chondrocytes that convert the effects of external forces to cellular responses. Diverse stimuli have been shown to exert chondroprotective effects, but our current knowledge is still incomplete and a better understanding of the molecular identity and function of mechanotransduction pathways is of crucial importance. It is very important to emphasize that the mechanical properties of native cartilage, and thus the responsiveness of chondrocytes to external stimuli, vary widely and depend on joint location, depth in the tissue, sample orientation, species, and donor age. These differences have important implications for cell-based regenerative approaches and should be considered during data interpretation. Further research should aim at understanding which load-induced biophysical changes are most important for cartilage ECM regeneration and maintenance of the chondrocyte phenotype to benefit functional cartilage tissue engineering.

## Abbreviations

ACI: Autologous chondrocyte implantation
ALP: Alkaline phosphatase
CCI: Characterized chondrocyte implantation
Cn: Calcineurin
CREB: cAMP response element binding protein
CsA: Cyclosporine A
ECM: Extracellular matrix
ERK: Extracellular signal-regulated kinase
FAK: Focal adhesion kinase
FCD: Fixed charge density
FTE: Functional tissue engineering
GAG: Glycosaminoglycan
HP: Hydrostatic pressure
MAPK: Mitogen-activated protein kinase
MSC: Mesenchymal stem cell
NFAT: Nuclear factor of activated T lymphocytes
OA: Osteoarthritis
OP: Osmotic pressure
PEMF: Pulsed electromagnetic field
PG: Proteoglycan
PKA: Protein kinase A
PKC: Protein kinase C
RVD: Regulatory volume decrease
TRPV: Transient receptor potential vanilloid channel
FF: Fluid flow
